# Change in GPS-assessed walking locations following a cluster-randomized controlled physical activity trial in older adults, results from the MIPARC trial

**DOI:** 10.1016/j.healthplace.2021.102573

**Published:** 2021-04-29

**Authors:** Katie Crist, Marta M. Jankowska, Jasper Schipperijn, Dori E. Rosenberg, Michelle Takemoto, Zvinka Z. Zlatar, Loki Natarajan, Tarik Benmarhnia

**Affiliations:** a Department of Family Medicine, UCSD, 9500 Gilman Drive, La Jolla, CA, 92093, USA; b Population Sciences, Beckman Research Institute, City of Hope, 1500 E Duarte Rd, Duarte, CA, 91010, USA; c University of Southern Denmark, J.B. Winsløws Vej 19, DK-5000, Odense C, Denmark; d Kaiser Permanente Washington Health Research Institute, Seattle, WA, USA; e Department of Psychiatry, UCSD, 9500 Gilman Drive, La Jolla, CA, 92093, USA; f Herbert Wertheim School of Public Health and Human Longevity Science, UC San Diego, La Jolla, CA, 92093, USA; g Scripps Institution of Oceanography, UCSD, 9500 Gilman Drive, La Jolla, CA, 92093, USA

**Keywords:** Aging, GPS, Life-space, Mobility patterns, Exercise/physical activity, Intervention

## Abstract

This study employed novel GPS methods to assess the effect of a multilevel physical activity (PA) intervention on device-measured walking locations in 305 community dwelling older adults, ages 65+ (mean age = 83, 73% women). Retirement communities were randomized to a 1-year PA intervention that encouraged neighborhood walking, or to a healthy aging control condition. Total time and time spent walking in four life-space domains were assessed using GPS and accelerometer devices. The intervention increased the time spent walking as a proportion of total time spent in the Campus, Neighborhood and Beyond Neighborhood domains. Intervention effects on walking location were observed in both genders and across physical and cognitive functioning groups. Results demonstrate that an intervention providing individual, social and environmental support for walking can increase PA in larger life-space domains for a broad spectrum of older adults.

## 1. Background

There is much interest in understanding the mobility patterns of older adults and developing interventions that can maintain health and independence across aging populations (Johnson et al., 2020; Matthews and Yang, 2013; Shareck et al., 2014; Taylor et al., 2019). Life-space and activity space measures describe the frequency and degree to which individuals move between geographically distinct domains that extend from within one’s home to beyond the neighborhood in which they live and have been related to mobility, physical activity (PA), falls, physical and cognitive function, nursing home admissions, and mortality in older adults (Baker et al., 2003; Hirsch et al., 2016; James et al., 2011; Johnson et al., 2020; Mackey et al., 2016; 2014; May et al., 1985; Peel et al., 2005; Portegijs et al., 2015; Schenk et al., 2011; Stalvey et al., 1999a; Takemoto et al., 2015; Taylor et al., 2019; H.-W Wahl et al., 2013). A larger life-space could also lead to more time spent outdoors, which has been linked to reduced loneliness, depressive symptoms, and fear of falling, as well as greater autonomy, PA and physical functioning (Abraham et al., 2010; Kerr et al., 2012a, 2012c; Portegijs et al., 2014; Rantakokko et al., 2014; Van Dyck et al., 2012). Globally, the older adult population is expected to double by 2050 (World Population Ageing, 2019 Highlights, 2019). Given this growth, PA interventions that target mobility in larger life-spaces could provide a valuable strategy to maintain health and autonomy in aging adults.

Retirement communities are an important housing option for the older adult population. Their campuses provide an additional life-space domain that allows residents to safely walk in well-maintained areas, without being exposed to neighborhood hazards outside of the property (Kerr et al., 2011). Most older adults, however, prefer to walk for a purpose and to a destination as opposed to simply walking for exercise (Winters et al., 2014; Yen IH, Shim JK, 2012) and walkable neighborhoods can support such purposeful activity (King et al., 2011). Life-space is typically assessed by self-report, though recent studies have successfully used mobile devices, like accelerometers and GPS, to measure life-space and out of home mobility objectively (Fillekes et al., 2019; Hirsch et al., 2016; 2014; Jansen et al., 2018; Liddle et al., 2014; Schenk et al., 2011; Shoval et al., 2011; Tsai et al., 2015; H.W. Wahl et al., 2013; Wan et al., 2013). Modifiable factors associated with life-space have been identified (Kuspinar et al., 2020) and studies have examined how the walkability of environments can moderate the effectiveness of PA interventions (Consoli et al., 2020; Kerr et al., 2010; Lo et al., 2019; Perez et al., 2018). However, no study has used validated GPS methods to investigate whether a PA intervention promoting neighborhood walking can increase walking in larger life-space domains.

The purpose of this study was to use accelerometer and GPS data from the Multilevel Intervention for Physical Activity in Retirement Communities (MIPARC) study to assess both the total time and *walking* time in four life-space domains (Home, Retirement Community Campus, Neighborhood, Beyond Neighborhood) in a 1-year PA intervention delivered in retirement communities. Since the ability to drive and access to transportation are strongly associated with larger life-spaces (Fillekes et al., 2019; Kuspinar et al., 2020; Shah et al., 2012), it is important to differentiate between active and passive (i.e. as a vehicle driver or passenger) time in different life-space domains. Previous findings from this cohort detected a significant increase and less decline in PA among the intervention group over time (Kerr et al., 2018). Since the intervention encouraged campus and neighborhood walking, we hypothesized that participants spent less time walking at home and more time walking in further life-space domains compared to the control condition participants. An exploratory objective was to examine whether the effect of the intervention on walking locations differed by gender, as we previously found that men in the intervention group had a greater increase in PA, compared to women (Kerr et al., 2018). We additionally explored whether baseline physical and cognitive function modified the effect of the intervention on walking location, as associations between these factors and mobility have been previously identified (Johnson et al., 2020; Kuspinar et al., 2020). We hypothesized that men and those with higher physical and cognitive functioning at baseline would have a greater increase in time walking beyond the Home and Campus domains, as a result of the PA intervention.

## 2. Methods

The institutional review board of the University of California, San Diego (UCSD) approved the study. All participants provided written informed consent and completed a post-consent comprehension test to rule out serious cognitive impairment. An independent data and safety monitoring officer reviewed all adverse events. The study was conducted according to the study protocol published elsewhere (Kerr et al., 2012b).

Using a cluster randomized study design, a total of 11 retirement communities were randomized to the intervention or attention control condition. Sites that met study criteria, agreed to either condition and signed a memorandum of understanding prior to being randomized. Eligible participants were at least 65 years old, completed a ‘timed up and go’ walking test in less than 30 s, were able to walk 20 m without human assistance, had not had a fall in the previous 12 months that resulted in hospitalization, were able to talk over the phone, and had no plans to move in the next year. The study was conducted between 2010 and 2014.

The intervention employed techniques from the Social Cognitive Theory and applied them in an Ecological framework with intervention activities occurring at the individual, interpersonal, and community level in and around the retirement communities (Bandura, 1986; Brawley et al., 2003; Sallis et al., 2006) as described previously (Kerr et al, 2012b, 2018). In addition to group education sessions and individual phone counseling, intervention participants received pedometers, educational materials, step counts for walking to common locations around their campus, and walking maps for their local community. The maps were created to reflect walks of varying lengths to different destinations in the neighborhood, e.g., parks or shops. At the start of the program, participants were encouraged to begin walking indoors and outdoors on their campus. By 6 weeks, neighborhood maps were distributed, and participants began group walks into the surrounding neighborhoods. The intervention was most intense in the first 6 months when all study components were co-led by UCSD staff and resident peer leaders, with the resident leaders continuing the walks during the subsequent 6-month follow up period. Resident leaders completed a walk audit with the pedestrian advocacy organization ‘Circulate San Diego’ and received training to advocate for improvement requests with local policy makers and city officials to ensure participants had safe walking routes in local neighborhoods. Community improvements included extending crosswalk times, adding auditory and visual traffic signals at busy intersections, clearing pedestrian paths of hazards, adding wheel stops to prevent cars from parking over a sidewalk, and adding walking paths to a retirement community redevelopment plan. Participants in the control condition received similar levels of attention via group meetings and counseling calls as intervention participants, though their sessions focused on topics related to successful aging, like nutrition and sleep, and the counseling calls asked about their general health. They did not receive pedometers, walking maps, nor any information related to PA or walking.

### 2.1. Measurement

Participants were measured at 5 time points: baseline, 3, 6, 9 and 12 months. Participants wore Qstarz GPS devices (BT-Q1000XT) attached to a belt worn on the waist, which can accurately differentiate indoor and outdoor time (Lam et al., 2013). Walking time was assessed using a triaxial accelerometer (GT3X+, ActiGraph) worn on the same belt as the GPS. At each measurement time point, participants wore both devices for 6 days during waking hours. Participants were asked to re-wear devices if they were not worn for a minimum of 10 h per day on at least 4 days. They were instructed to charge the GPS device overnight.

The GPS data were processed and joined to the accelerometer data using the Personal Activity and Location Measurement System (PALMS) (Carlson et al., 2015). Data were aggregated and merged at the minute level. For this study, activity was classified into intensity defined categories of walking (≥760 counts per minute (cpm)) and not walking (<760 cpm). The 760 cpm threshold has been shown to accurately measure moderate to vigorous intensity activities in older adults (Matthews, 2005; Matthews et al., 2013; Rejeski et al., 2016). The intervention targeted an increase in daily step counts, and did not focus on the intensity of walking, thus this threshold was deemed most appropriate to capture all walking, especially given the older age of the study population. Non-wear time was determined using a modified Choi algorithm in which 90 consecutive minutes of zero counts with a 2-min spike tolerance was screened as non-wear (Choi et al., 2011). Missing GPS data was imputed using a validated algorithm (Meseck et al., 2016). After consideration of non-wear time and missing GPS data, valid wear days were defined as days with a minimum of 600 min of combined accelerometer and GPS data.

### 2.2. Time in life-space domains

**Fig. 1.**
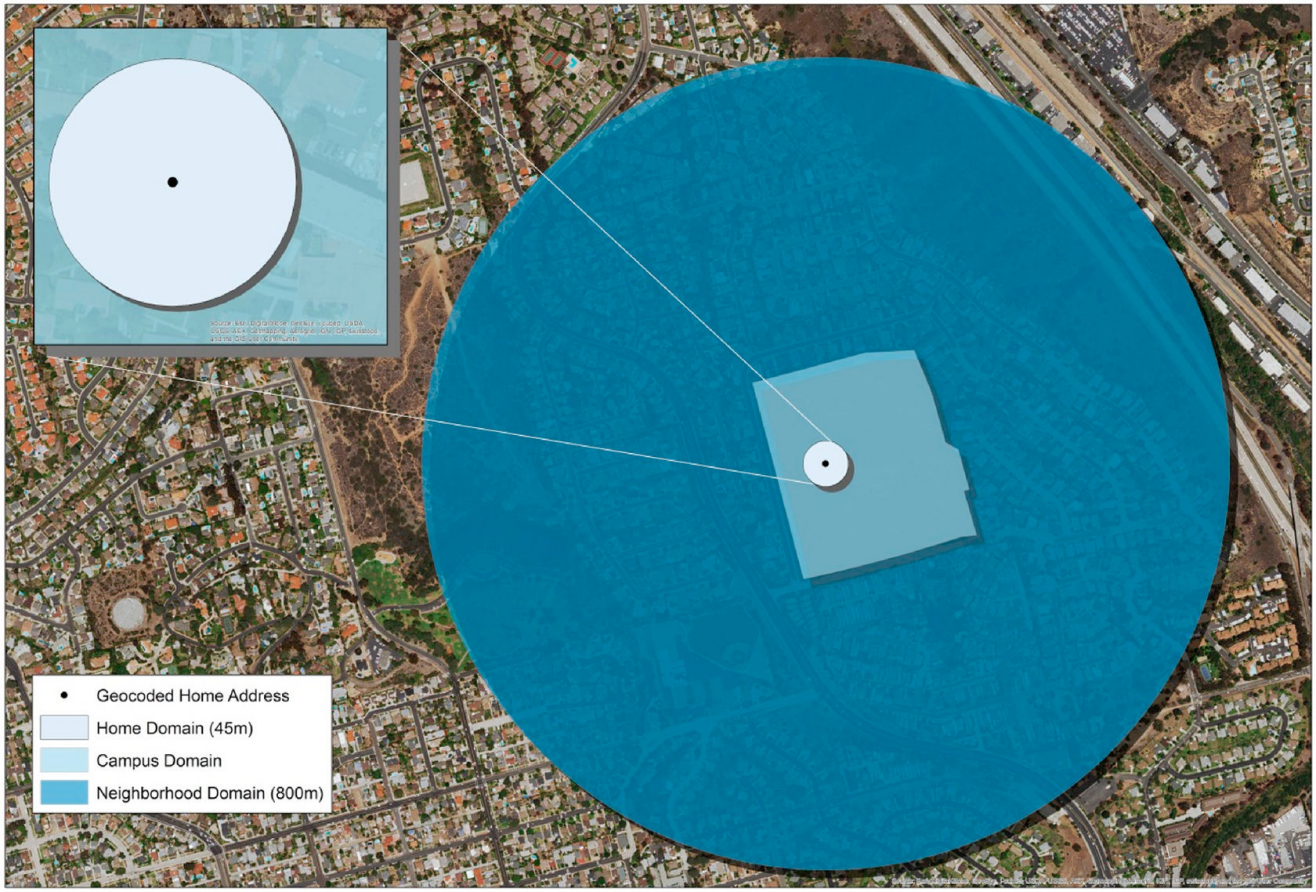
Example of mutually exclusive life-space domains: Home, Campus, Neighborhood, and Beyond Neighborhood.

Four domains were defined: Home, Retirement Community Campus (Campus), Neighborhood, and Beyond Neighborhood to represent the original life-space survey (Baker et al., 2003). Home address for each participant was determined by taking the centroid mean of all GPS coordinates at 3am for each day. This calculation was performed to give a precise home location on large campuses, rather than using a retirement community street address, which would generalize all participants for a given retirement community to one geocoded point at the street level. The Home domain was defined spatially by creating a 45 m radial buffer around the participant’s home location to account for the home footprint as well as a small GPS scatter buffer. The Campus domain was defined manually using satellite imagery to create a polygon outline of each of the MIPARC site campuses with the accuracy reviewed by a UCSD staff member familiar with each site. The Neighborhood domain was defined spatially by creating an 800 m radial buffer from each participant’s home location, similar to a study using smartphones to assess life-space (Wan et al., 2013). Neighborhood is commonly defined by a 10–20 min walk in older adult studies (Barnett et al., 2017). In this sample, the average time to complete a 400 m walk test (Vestergaard et al., 2009) was 7.4 min, thus we estimated that an 800 m buffer would meet the 10–20 min neighborhood definition, a distance supported by prior research (Boruff et al., 2012; Mavoa et al., 2019). The Beyond Neighborhood domain was defined as any point beyond the 800 m Neighborhood buffer. While many built environment studies use local street network buffers, street networks typically do not include sidewalks and other formal or informal walking paths, so radial buffers were employed to capture all space in relatively close proximity to the participants’ home and to effectively model the mutually exclusive life space framework (Fig. 1).

GPS, accelerometer, and spatial domain data were loaded into a HIPAA compliant PostgreSQL geodatabase. Python and SQL commands were used to spatially join each GPS and accelerometer point to a life-space domain, and participant data were aggregated to average total daily minutes and total daily walking minutes in each domain for each time point. We calculated percent total time (time in each domain out of total awake time) and percent time spent walking in each domain (minutes walking in a domain out of total time in the domain) for each participant.

### 2.3. Covariates

Age, gender, education level (college and above vs. less than college) and marital status (married/not married) were assessed by self-report. Baseline physical and cognitive functioning were assessed as potential effect modifiers as both continuous and categorical variables. The Short Physical Performance Battery (SPPB) was used to objectively measure physical function as it is predictive of disability (Guralnik et al, 1994, 2000). The total score was calculated (possible range 0–12) and a dichotomous variable created to indicate low (0–9) or high (10–12) functioning (Bandinelli et al., 2006; Guralnik et al., 1994).

Cognitive function was measured using the Trail Making Test (TMT) – Parts A and B, which assesses cognitive flexibility and executive function, and the Wechsler Adult Intelligence Scale – IV Symbol Search Test, to assess visual scanning and psychomotor speed. Both have been shown to vary with PA (Barha et al., 2017; Chen et al., 2018; Sanders et al., 2019; Vidoni et al., 2015; Zlatar et al., 2019). The TMT was scored in time (seconds) to completion with higher scores representing worse cognitive functioning. The Symbol Search Test allowed 120 s for completion and higher scores were indicative of better functioning. We averaged z-scores from all 3 cognitive tests to create a composite score. Trails A and B scores were multiplied by −1 so that a positive score would represent better cognition, thus higher scores on the cognitive composite reflect better cognitive performance. We also split the composite score into 3 categories: “low” indicated a composite score ≤ −1 standard deviation (SD) from the group mean (n = 23, 10%), “middle” between −1 and +1 SD (n = 186, 81%), and “high” ≥ +1 SD (n = 20, 9%) and tested effect modification using both continuous and categorical variables.

### 2.4. Statistical analyses

The study sample was described using means for continuous variables and frequencies for categorical variables, with *t*-test or chi square tests assessing statistical differences between intervention and control groups at baseline. We quantified the difference in outcomes by intervention status across time using mixed effects linear regression models. First, we assessed change in daily time (min/day) in non-home domains (i.e., sum of Campus, Neighborhood and Beyond Neighborhood time), total daily walking (across all domains) and walking in Non-home domains. The total time and time spent walking (both as daily minutes and as a proportion of time spent in the domain) were analyzed as separate outcomes for each life-space domain. Multiple measurement days were nested within participants, and a random participant-level intercept was included in the model. Condition (intervention versus control), time, and a two-way interaction effect, condition x time (i.e., the intervention effect), were included as fixed effects. To account for retirement community clustering, study site was entered as a fixed effect.

We obtained and plotted the marginal effect for each measurement time point by condition, which averages the predicted values of the dependent variables by time point and condition for all observations, accounting for all other covariates (Williams, 2012). Three-way interactions assessed whether the intervention impact on walking in the 4 life-space domains differed based on gender or baseline physical and cognitive function, using both continuous and categorical physical and cognitive functioning variables in separate models to test sensitivity. Categorical variables were used in margins plots to aid visualization. All analyses were conducted in Stata SE 14.2 (StataCorp, College Station, TX).

### 2.5. Inverse probability of treatment weighting (IPTW)

Randomization occurred at the retirement community level and, as is common when the number of clusters is low, randomization did not achieve balance across all covariates at the participant level (Moerbeek, 2005; Moerbeek and Van Schie, 2016). We used IPTW to adjust for covariate imbalance between the intervention and control groups at baseline and any residual confounding. We first modeled participants’ probability of being in the intervention group (i.e. the propensity score (PS)) (Leyrat et al., 2013). We considered baseline demographic characteristics, many of which were imbalanced (age, gender, education, marital status, baseline PA, physical and cognitive functioning) (Amaducci et al., 1998; Leveille et al., 2000; Matthews et al., 2005), or covariates that were thought to potentially influence the outcomes as follows (Brookhart et al., 2006; Rosenbaum and Rubin, 1983). The Falls Efficacy Scale - International (FES-I) assessed fear of falling, which has been associated with PA and outdoor time in older adults (Kempen et al., 2008; Kerr et al., 2012a; Rantakokko et al., 2009). Self-reported functional performance and disability was assessed using the Late Life Functioning Disability Instrument (LLFDI) (Beauchamp et al., 2014; Sayers et al., 2004). The 6 item short form of the PROMIS Pain Interference Scale assessed the degree to which pain impacted life activities (Amtmann et al., 2010). The time to complete the 400 Meter Walk Test (MWT) assessed cardiorespiratory fitness (Anton et al., 2011). Device-based covariates included accelerometer-assessed average daily moderate-to-vigorous PA (MVPA) minutes (≥1952 counts per minute), sedentary minutes (<100 counts per minute) and device wear time (mins/day) at baseline. The categorical physical and cognitive functioning variables were also included in the PS.

A weight was then calculated using the PS values and included in regression models. IPTW removes confounding by creating a pseudopopulation in which every participant has an equal probability of receiving the intervention and control. Standardized Mean Differences (SMD) for each covariate included in the PS were computed to assess balance between the intervention and control groups before and after weighting. A threshold of SMD <0.1 was used to determine acceptable balance (see Appendix 1) (Austin and Stuart, 2015) and to choose which covariates to include in the final PS model to calculate weights. The final weighted outcome regression models additionally adjusted for age, gender, marital status, education, study site and categorical baseline physical and cognitive function variables, making our estimation doubly robust.

## 3. Results

There were 305 participants in total with 150 and 155 participants in the intervention and control arms, respectively. Table 1 provides descriptive statistics for the MIPARC sample at baseline. Participants in the intervention arm were younger, more likely to be married, and had higher physical function scores. Intervention participants spent less time at Home and Campus and more time in the Neighborhood and Beyond at baseline. On average, intervention participants had more daily walking at baseline, with more minutes walking in the Neighborhood and Beyond Neighborhood domains, compared to controls.

**Fig. 2.**
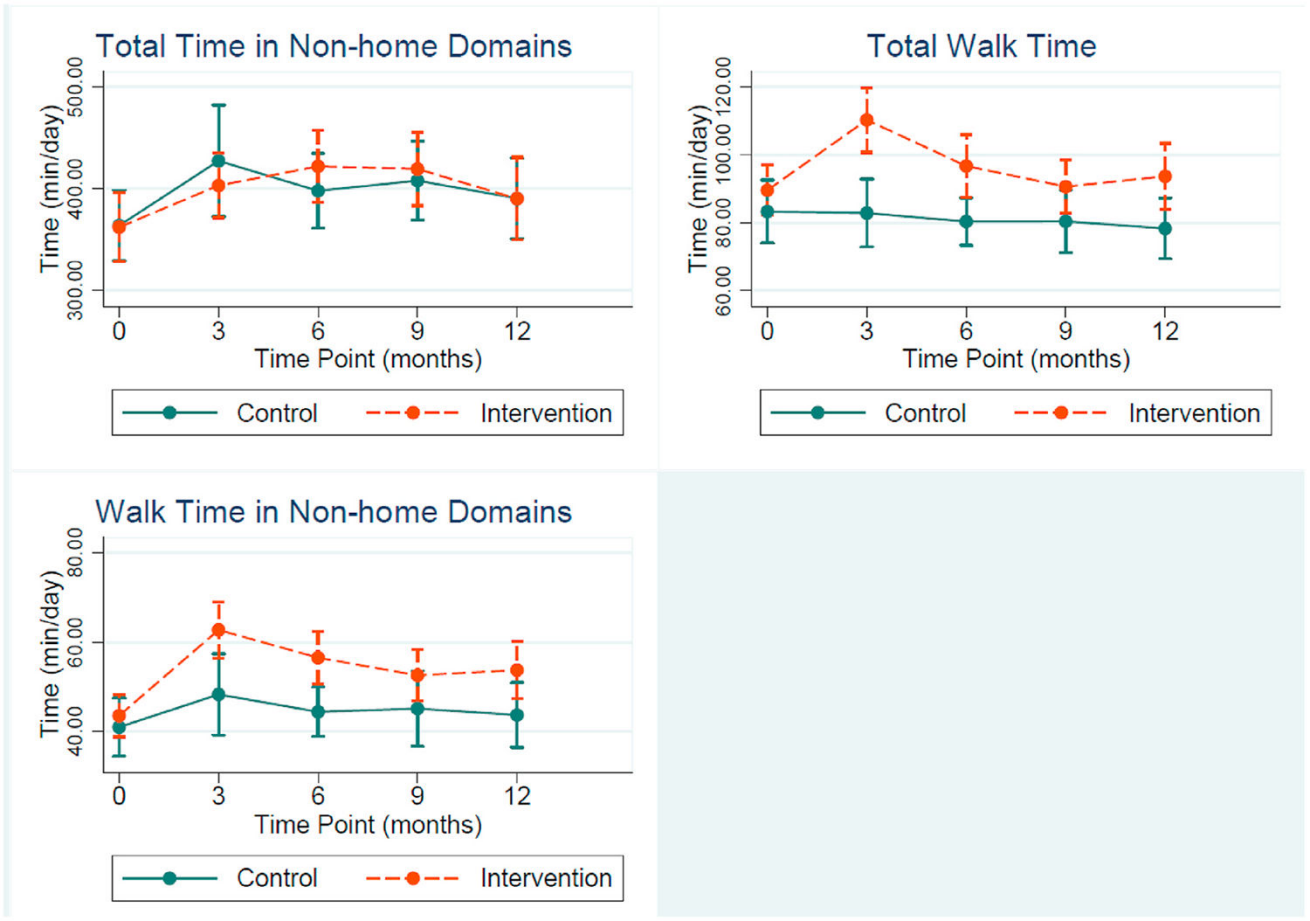
Marginal estimates and 95% confidence intervals for time in non-home domains, total walk time and walk time in non-home domains (min/day).

Results of the mixed effects linear regression models are presented as plots of the marginal estimates by condition across study time points in Fig. 2. Confidence intervals on the margin plots indicate precision of the estimates at that time point, whereas estimates of the condition by time interaction are presented with regression coefficients and 95% confidence intervals in the appendices. Overall, we did not observe any intervention effect on overall time spent in non-home domains (Fig. 2). The intervention group increased their daily walking from baseline to 3 months by 21.48 min/day (CI: 12.0, 31.0, Appendix 2), compared to controls, and maintained greater daily walking levels throughout the remainder of the study, whereas there was no change in walk time among the control group (Fig. 2). A significant increase in daily time spent walking in non-home domains was also observed among intervention participants at 3 months (coef = 11.48, 95% CI: 1.7, 21.3), and remained higher, compared to controls, though estimates were imprecise (Appendix 2).

**Fig. 3.**
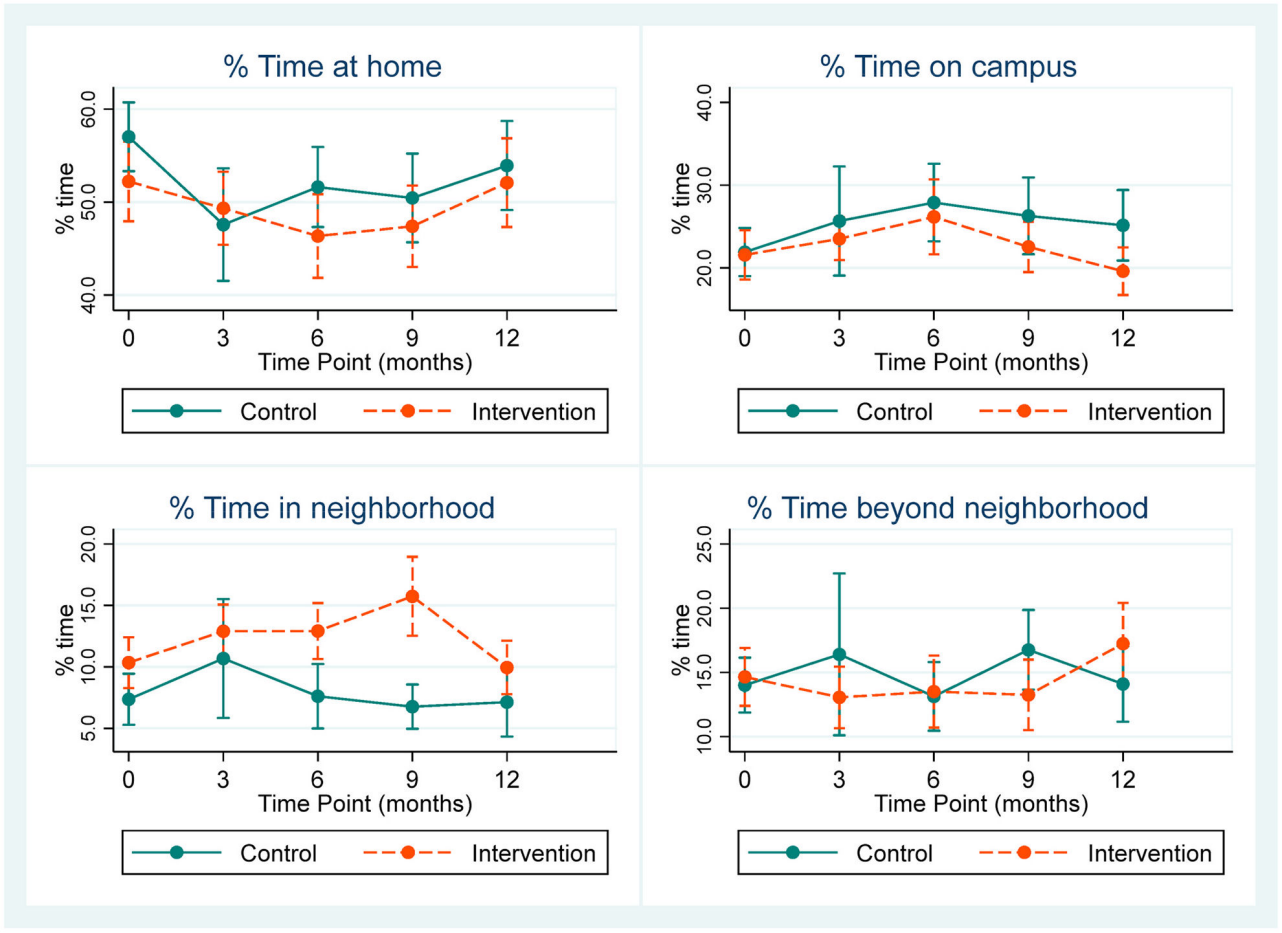
Marginal estimates and 95% confidence intervals for percentage of time spent in life-space domains.

Fig. 3 shows marginal estimates of the proportion of time spent in each life-space domain (out of overall awake time). We did not observe differences between groups in the percent of time spent in any life-space domain, except that intervention participants had greater time in the neighborhood domain at 6 and 9 months, compared to control participants. In general, there were not any significant differences in trends over time between groups (Appendix 3).

**Fig. 4.**
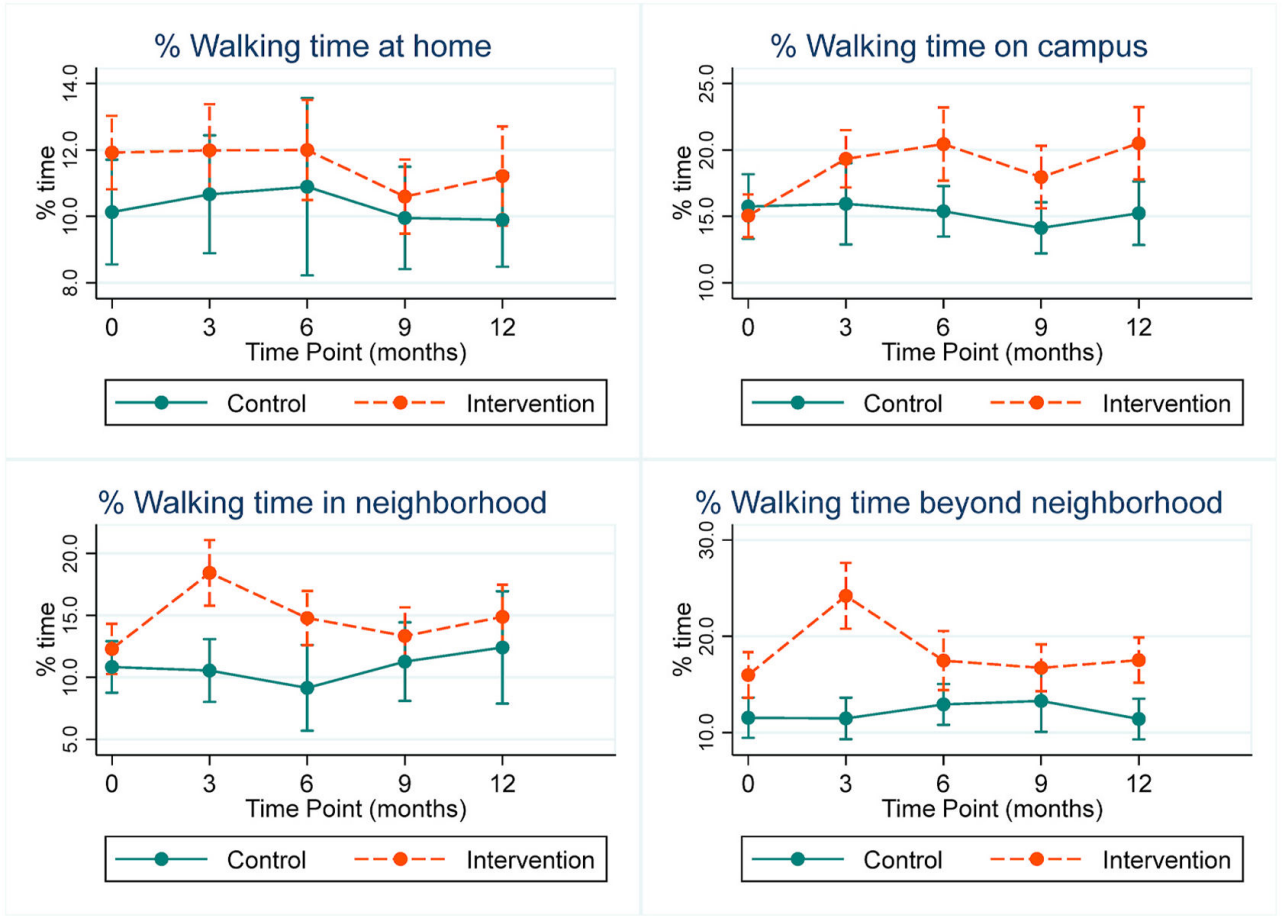
Marginal estimates and 95% confidence intervals for percentage of time spent walking in life-space domains.

Out of the total time spent in each domain, intervention participants spent a greater percentage of time walking than controls across all domains and time points, though baseline differences between groups were imprecise in most domains (Fig. 4). The most sustained change occurred in the Campus domain where intervention participants increased their percent walking time from baseline at all time points, compared to controls (Appendix 4). The intervention group had a large increase in percent time spent walking in both the Neighborhood and Beyond Neighborhood (6% and 8%, respectively) domains at 3 months (Appendix 4), with differences remaining at 12 months in Beyond Neighborhood walking. See Appendix 5 for marginal estimates and 95% confidence intervals for % time walking in life-space domains.

**Fig. 5.**
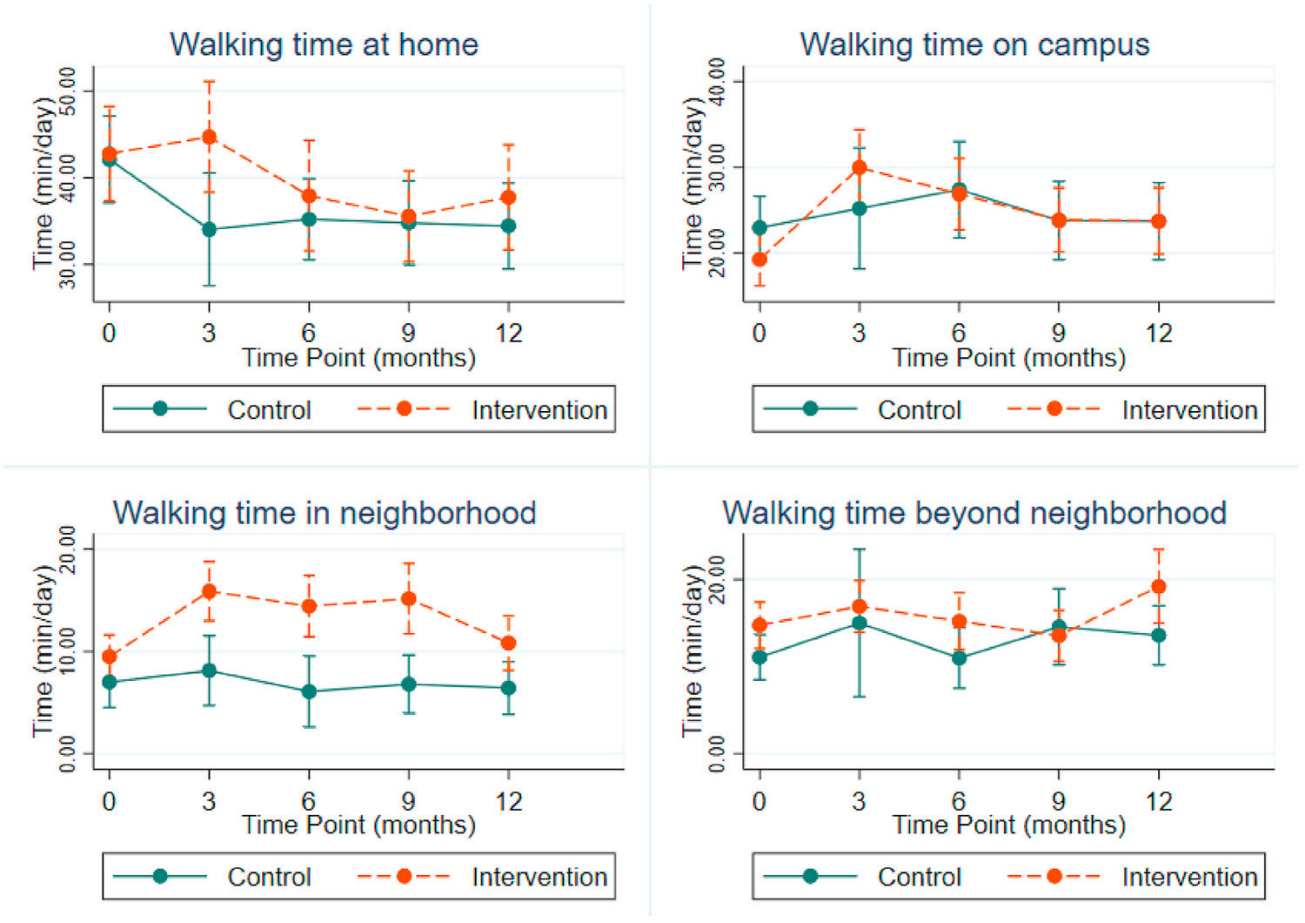
Marginal estimates and 95% confidence intervals for time spent walking in life-space domains (min/day).

We additionally assessed total minutes walking in each domain to explore how each domain contributed to overall walking time (Fig. 5). Most walking, in absolute minutes, occurred in the Home and Campus domains. The intervention group had greater walking minutes in the Neighborhood at 3, 6 and 9 months, compared to controls, whereas differences between groups were not observed at any time point in other domains. Regression outputs and marginal estimates for minutes of walking by time point and condition are presented in Appendices 6 and 7.

We did not observe notable differences in the intervention effect on percent time walking by gender or physical and cognitive function levels. In the intervention group, we found greater Beyond Neighborhood walking by females at 3 months while men had more Campus walking at 6 months, however interaction term coefficients were imprecise and general trends were similar by gender (Appendix 8).

In general, we observed a greater percentage of time spent walking by those with better physical function in both intervention and control participants (Appendix 9). Intervention participants with both low and high physical function had a trend of increased walking that was maintained over time in the Campus, Neighborhood and Beyond Neighborhood, compared to controls. Those with low function returned to near baseline levels by 12 months in the furthest domains, though maintained higher campus walking. The high functioning group sustained walking gains over the 12-month period, especially in the Campus and Neighborhood domains.

Generally, intervention participants with the highest cognitive function had more walking than the other cognitive groups in non-home domains, though the mid and low functioning groups also showed an increase in walking outside the home at 3 months (Appendix 10). Among controls, the high cognition group also generally had more walking than other groups, though the pattern was less clear.

## 4. Discussion

Our main finding was that intervention participants increased their total walking in the Campus, Neighborhood and Beyond Neighborhood domains, compared to the control group. The intervention did not appear to affect overall time spent in life-space domains. These results indicate that the intervention was effective specifically in increasing PA in further life-space domains.

Increased walking as a percentage of Campus time was sustained by intervention participants across the entire 12-month period, while walking in the Neighborhood and Beyond Neighborhood domains had the greatest increase at 3 months and then declined over time. The termination of UCSD staff’s role in the intervention after 6 months may have contributed to these observed declines. Intervention participants did spend more time, both walking and in general, in the Neighborhood domain than the control group. It may be that the increased exposure to neighborhood destinations (i.e. new parks or shops) gained in the walking intervention led participants to increase time there, both for walking or other activities (Kerr et al., 2012a; Winters et al., 2014).

We did not find that men in the intervention group increased their walking in further life-space domains more than women, as hypothesized. These findings are unexpected given that we observed a greater increase in MVPA among men in a separate analysis of this cohort (Kerr et al., 2018). It may be that men increased the proportion of their overall activity that was at higher intensity (i.e., MVPA), but the location of that activity did not differ. Studies have found gender differences in environmental perceptions and confidence to walk in local environments, with women walking less in neighborhoods (Dyck et al., 2012; Gallagher et al., 2014; Merom et al., 2015). The social support and group walks provided by the intervention may have been successful in overcoming the barriers experienced by women that lead to these differences.

One unexpected finding was that in the Neighborhood and Beyond Neighborhood domains, both those with high and low physical function had a large increase in walking at 3 months that tapered off, though more drastically in the lower functioning group. This is an important finding given that, in baseline analyses in this cohort, we found the frequency, distance and duration of pedestrian trips were positively associated with better physical functioning (Takemoto et al., 2015). Our findings suggest that programs providing individual, social and environmental support for walking may mitigate the decline in walking and life-space mobility that may otherwise occur due to low physical functioning, though future studies should explore how to maintain gains over a longer term.

In line with results from this analysis, we did not find cross-sectional associations between cognitive function and walking in prior analyses of this cohort (Takemoto et al., 2015). Nor were associations found in a 2-year study assessing cognitive decline and life-space mobility (Béland et al., 2018). A recent review found evidence that those with limited life-spaces were more likely to develop mild cognitive impairment (De Silva et al., 2019), which we did not assess. That said, we found that intervention participants with higher cognitive function spent more time walking in non-home domains, compared to the other cognitive groups. This suggests that individuals with better cognitive function may benefit more from PA interventions that target walking in further life-space domains while more specific intervention strategies would be needed for a more cognitively impaired sample to ensure comfort and safety with walking further into their surrounding environment. This is important to explore given that, for older adults with cognitive decline, maintaining out-of-home life-space has been shown to positively impact satisfaction and independence as well as a sense of autonomy and identity (Kerr et al., 2012a; Portegijs et al., 2014; Rantakokko et al., 2014).

Our study is the first to use GPS to assess changes in where individuals walk, based on an intervention that addressed the walkability of local environments. Webber et al. suggested that the determinants of older adult mobility are influential at the individual, social and environmental levels (Webber et al., 2010). Our lack of observed effect modification by physical or cognitive function align with results from a recent large study that showed driving status, social support, and gait speed were the most significant determinants of life-space mobility, while balance and cognitive factors, like memory and executive function, were less important (Kuspinar et al., 2020). Our study demonstrates that a multilevel intervention to increase PA, that includes a focus on both supportive social and built environments, can increase PA in larger life-space domains, at least temporarily. Future research is needed to understand how to maintain mobility gains, especially in more vulnerable groups.

While this study had many strengths, including a novel multilevel intervention and device-measured PA and location, the small number of retirement communities resulted in lower statistical precision and environmental variability. Accessible life-space may have varied since some sites were close to the ocean and we did not test the sensitivity of the neighborhood buffer size. Studies should further investigate what environmental predictors may affect walking in life-space domains. The two most common life-space assessments in community dwelling populations range from 3 days to 4 weeks, whereas our measure was for one week (Baker et al., 2003; Stalvey et al., 1999b). Though shorter in duration than the University of Alabama Life-Space Assessment (UAB-LSA), self-reported measures require more recall, which may be difficult in an older adult population. The combination of GPS and accelerometer data provide an objective measure of frequency, duration and PA in various life-space domains. However, some questionnaires additionally capture the degree of independence when traveling in life-space domains. While the study population was ambulatory without human assistance, participants may have used assistive devices for walking, which was not considered in this analysis. Further, we did not attempt to generate a single life-space mobility score, as is the goal with questionnaires, though this could be explored in future work. Though driving, a key life-space determinant, was not specifically addressed, this cohort had access to transportation for recreation and shopping trips through their retirement communities that likely mitigated the importance of this factor. Our cognition classification was based on a limited set of assessments and we had very few participants with high or low scores, which may have limited our ability to detect any moderating effects, though no effect was observed using either the continuous or categorical cognition score.

## 5. Conclusions and implications

In community dwelling residents, maintaining life-space mobility is critically important to avoid physical and cognitive decline, risk of falls, and possible transition to a care facility. Maintaining independence and PA may help to reduce healthcare costs, living costs, and give elderly residents a greater sense of autonomy. While maintaining older adults’ life-space mobility can be supported by medications, vision and hearing tests, and transportation options, our results indicate a walking program could increase PA in larger life-space domains, providing the additional physical and psychological health benefits associated with PA and outdoor time. There was no difference in the number of adverse events between intervention and control participants, so participants remained safe despite walking in more challenging environments.

This study provides evidence that mobility patterns can be changed, even in quite elderly adults, through a targeted intervention with strategies specifically aimed at providing social support and introducing new walking environments. These elements may explain why the effect of the intervention did not differ by gender, physical function and cognition as expected, indicating that the many older adults, regardless of functional status, may benefit. Campus was the only domain where walking continued to increase across the length of the study period, highlighting the importance of those spaces that are most accessible to home locations. The findings that the largest increases occurred at 3 months followed by a decline highlight the importance of developing effective ongoing intervention strategies.

## Figures and Tables

**Table 1.** MIPARC participant characteristics at baseline.

	Intervention	Control	p- value
N	150	155	
Age (years), mean (SD)	81.3 (5.8)	84.8 (6.5)	0.000
Female, n (%)	111 (74.0)	110 (71.0)	0.550
College education or above, n (%)	99 (66.0)	93 (60.0)	0.110
Married, n (%)	76 (50.7)	46 (29.7)	0.000
Total time in each domain, mean min/day (SD)			
Home	428.2 (254.9)	488.0 (231.4)	0.000
Campus	157.8 (169.8)	178.3 (168.7)	0.018
Neighborhood	85.6 (151.5)	65.1 (131.4)	0.005
Beyond neighborhood	113.0 (159.0)	87.4 (136.1)	0.001
Total walking time, mean min/day (SD)	83.8 (45.4)	72.7 (48.0)	0.000
Total walking time in each domain, mean min/day (SD)			
Home	40.6 (34.5)	38.2 (29.6)	0.150
Campus	17.9 (21.1)	19.9 (22.7)	0.072
Neighborhood	9.4 (18.3)	5.6 (13.4)	0.000
Beyond neighborhood	13.5 (25.6)	8.1 (16.3)	0.000
Daily device wear time, mean min/day (SD)	808.7 (78.3)	825.3 (75.9)	0.000
Physical function (SPPB), mean (SD)	9.4 (2.5)	8.2 (2.7)	0.000
Cognition composite z-score, mean (SD)	0.2 (0.8)	−0.1 (0.8)	0.000

* p-values from *t*-test or chi square tests.

**Appendix 8.**
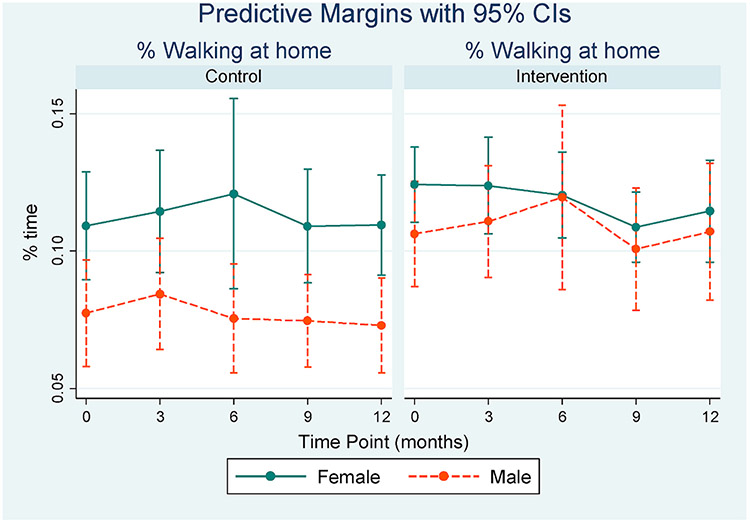

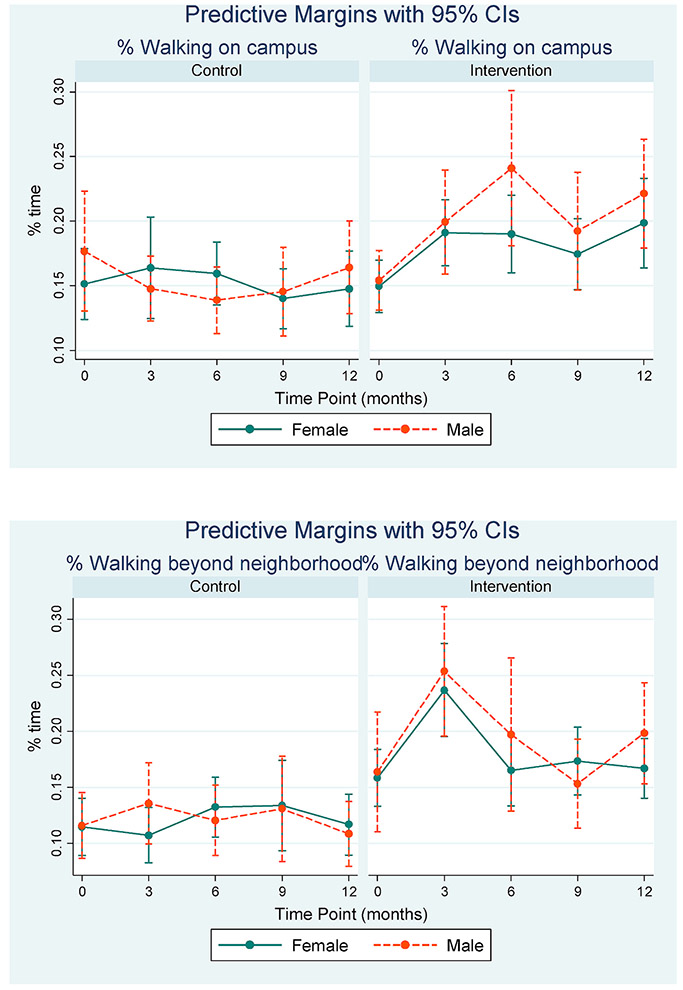

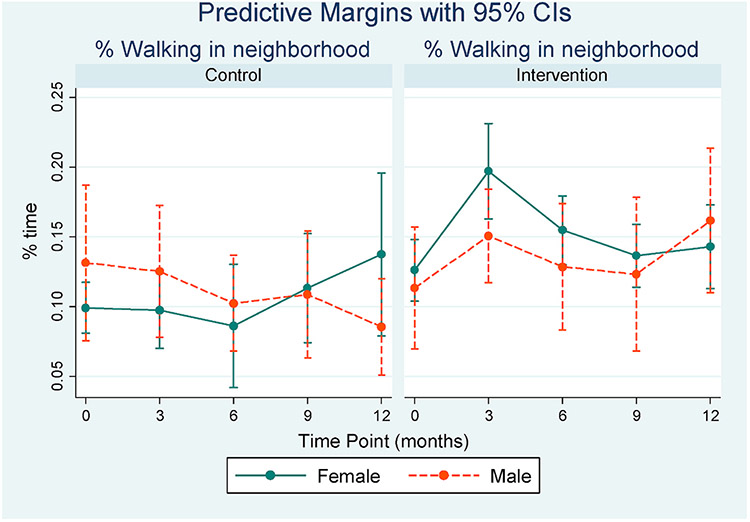
Marginal estimates and 95% confidence intervals for percentage of time spent walking in life-space domains for condition x time × gender interaction

**Appendix 9.**
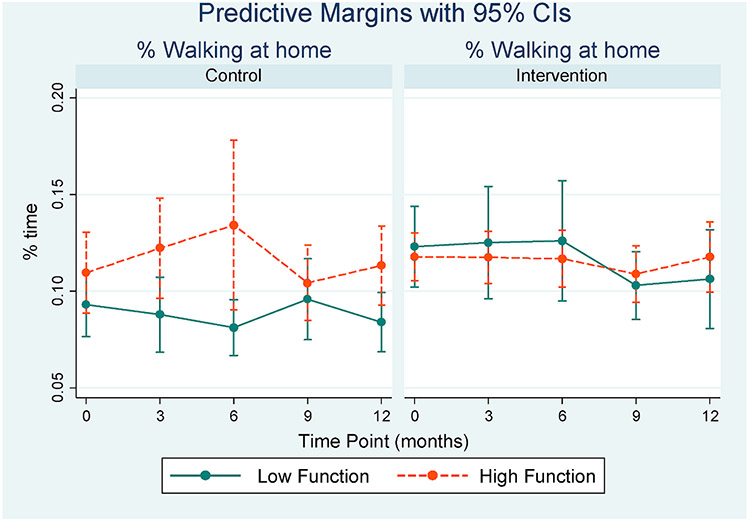

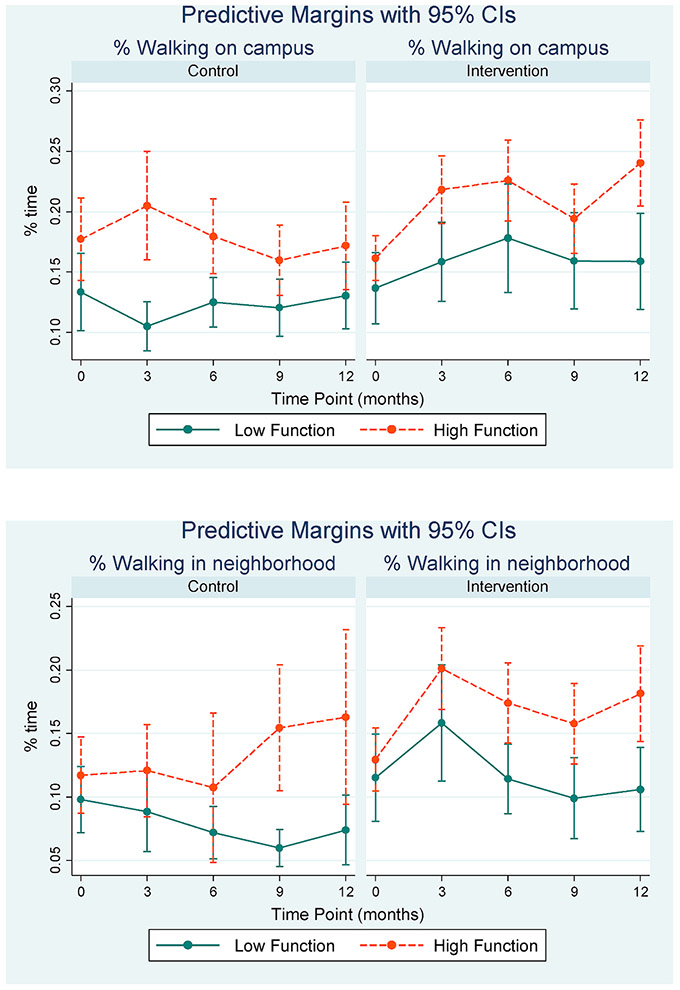

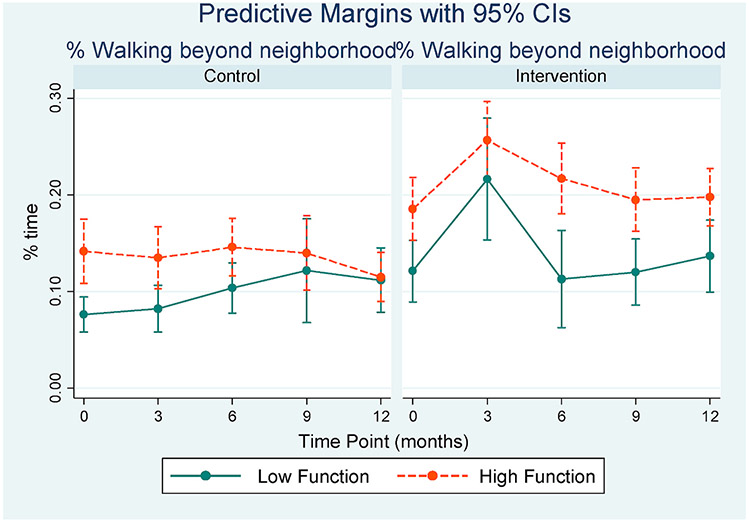
Marginal estimates and 95% confidence intervals for percentage of time spent walking in life-space domains for condition x time x physical function interaction

**Appendix 10.**
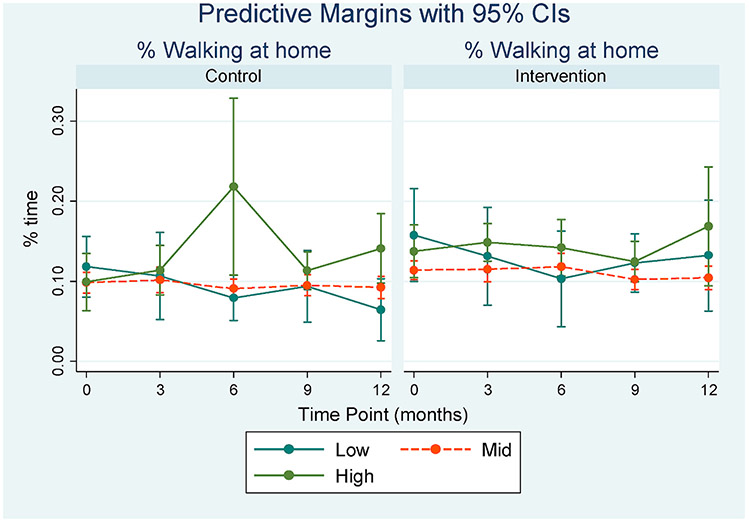

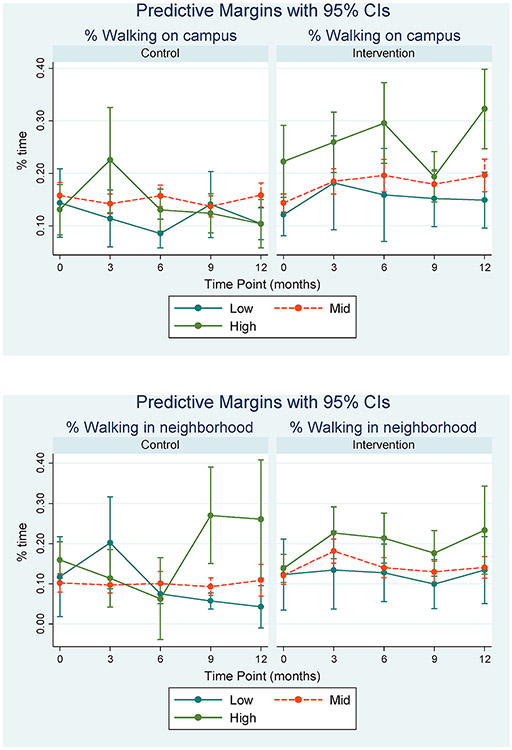

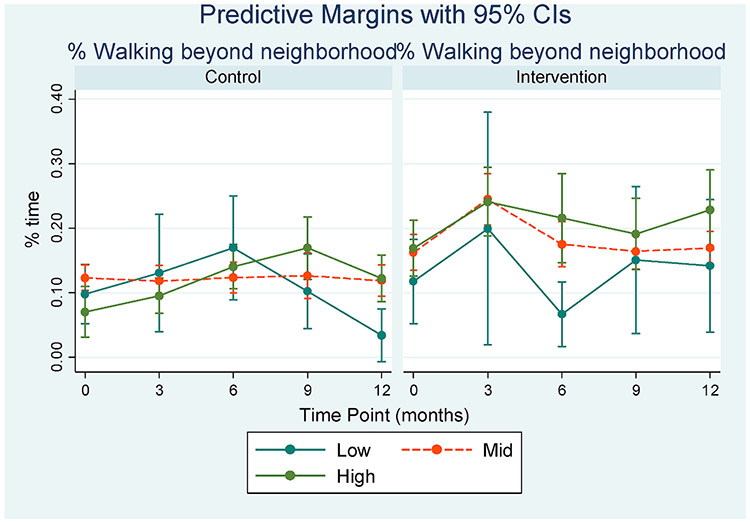
Marginal estimates and 95% confidence intervals for percentage of time spent walking in life-space domains for condition x time × cognition interaction

## Appendix

**Appendix 1.** Standardized Mean Differences (SMD) of propensity score covariates between intervention and control groups, before and after weighting

	SMD	SMD
	Before Weighting	After Weighting
Age	−0.59437	−0.00205
Gender	−0.20831	0.022834
Education	0.089714	0.023482
Marital status	0.258244	−0.01542
Avg. daily wear time	−0.19829	−0.03121
Average daily MVPA	0.292411	0.030082
Physical function (SPPB)	0.473672	0.003744
Composite cognitive score	0.410389	−0.03931
Age x SPPB	0.411539	0.014921
Age x gender	−0.22189	0.017945
Fear of falling	−0.42693	−0.08352
Fear of falling x age	−0.53473	−0.07322
Avg. daily sedentary time	−0.38379	−0.025
Disability	0.522758	−0.03109
Pain	−0.17361	0.065103
400 MWT	−0.54127	0.084759

**Appendix 2.** Regression estimates for non-home time, total walking time and walking time in Non-home domains (min/day)

	Non-home time		Total walk time		Non-home walking	
	Coef	95% CI	coef	95% CI	coef	95% CI
Intervention condition	−17.4	[−192.5,157.8]	−10.1	[−36.2,16.1]	−9.0	[−24.3,6.3]
Time point (Ref)						
3 mos	60.3*	[1.0,119.5]	−1.1	[−7.4,5.3]	6.7	[−0.9,14.3]
6 mos	36.0*	[0.2,71.8]	−2.0	[−9.2,5.1]	4.1	[−2.7,10.8]
9 mos	54.9*	[15.9,94.0]	−0.3	[−11.5,10.9]	6.4	[−4.9,17.7]
12 mos	29.8	[−4.4,64.0]	−4.1	[−13.8,5.6]	3.1	[−6.0,12.3]
Condition x time						
Intervention x 3 mos	−26.2	[−93.0,40.7]	21.5*	[12.0,31.0]	11.5*	[1.7,21.3]
Intervention x 6 mos	9.4	[−38.0,56.8]	8.5	[−1.6,18.6]	7.3	[−1.5,16.1]
Intervention x 9 mos	−14.3	[−65.5,36.8]	0.8	[−12.0,13.6]	0.9	[−11.5,13.3]
Intervention x 12 mos	−11.9	[−64.7,40.9]	8.0	[−4.5,20.5]	6.1	[−4.7,16.9]

95% confidence intervals in brackets.

* *p* < 0.05.

**Appendix 3.** Regression estimates for percent time spent in each domain out of total awake time

	Home		Campus		Neighborhood		Beyond Neighborhood	
	coef	95% CI	coef	95% CI	coef	95% CI	coef	95% CI
Intervention condition	0.01	[−0.22,0.23]	−0.16	[−0.32,0.00]	0.03	[−0.02,0.08]	0.13*	[0.02,0.24]
Time point (Ref)								
3 mos	−0.09*	[−0.16,−0.03]	0.03	[−0.04,0.10]	0.04	[−0.02,0.10]	0.02	[−0.05,0.09]
6 mos	−0.06*	[−0.10,−0.02]	0.05*	[0.02,0.09]	0.01	[−0.01,0.03]	−0.00	[−0.03,0.02]
9 mos	−0.08*	[−0.12,−0.04]	0.03	[−0.01,0.08]	0.01	[−0.01,0.03]	0.04*	[0.01,0.07]
12 mos	−0.04	[−0.08,0.00]	0.02	[−0.01,0.06]	0.01	[−0.02,0.04]	0.00	[−0.03,0.04]
Condition x time								
Intervention x 3 mos	0.07	[−0.01,0.14]	−0.00	[−0.08,0.07]	−0.02	[−0.08,0.04]	−0.04	[−0.12,0.03]
Intervention x 6 mos	0.01	[−0.04,0.07]	0.00	[−0.05,0.06]	−0.00	[−0.03,0.03]	−0.01	[−0.06,0.03]
Intervention x 9 mos	0.05	[−0.01,0.11]	−0.01	[−0.07,0.04]	0.03	[−0.01,0.07]	−0.06*	[−0.11,−0.02]
Intervention x 12 mos	0.05	[−0.01,0.11]	−0.04	[−0.09,0.01]	−0.03	[−0.06,0.01]	0.02	[−0.03,0.06]

95% confidence intervals in brackets.

* *p* < 0.05.

**Appendix 4.** Regression estimates for percent time walking in each domain out of total time in the domain

	Home		Campus		Neighborhood		Beyond Neighborhood	
	coef	95% CI	coef	95% CI	coef	95% CI	coef	95% CI
Intervention condition	0.02	[−0.02,0.06]	−0.01	[−0.10,0.07]	−0.01	[−0.07,0.04]	−0.05	[−0.13,0.03]
Time point (Ref)								
3 mos	0.01	[−0.01,0.02]	0.00	[−0.04,0.04]	−0.00	[−0.03,0.02]	−0.00	[−0.02,0.02]
6 mos	0.02	[−0.03,0.06]	−0.00	[−0.02,0.02]	−0.02	[−0.05,0.02]	0.01	[−0.01,0.04]
9 mos	0.01	[−0.00,0.02]	−0.01	[−0.03,0.01]	0.01	[−0.03,0.04]	0.02	[−0.02,0.06]
12 mos	0.00	[−0.01,0.02]	−0.01	[−0.02,0.01]	0.02	[−0.03,0.06]	0.00	[−0.03,0.03]
Condition x time								
Intervention x 3 mos	−0.01	[−0.03,0.01]	0.04	[−0.01,0.09]	0.06*	[0.02,0.10]	0.08*	[0.04,0.12]
Intervention x 6 mos	−0.02	[−0.07,0.02]	0.05*	[0.02,0.09]	0.04	[−0.00,0.08]	−0.00	[−0.04,0.04]
Intervention x 9 mos	−0.02*	[−0.04,−0.01]	0.04*	[0.01,0.07]	0.00	[−0.04,0.05]	−0.01	[−0.06,0.04]
Intervention x 12 mos	−0.01	[−0.04,0.01]	0.06*	[0.03,0.09]	0.01	[−0.04,0.06]	0.01	[−0.03,0.05]

**Appendix 5.** Marginal estimates and 95% confidence intervals for % time walking in life-space domains

Time Point x Condition	Marginal estimate (%)	95% CI
**Home domain**		
Baseline Control	0.101	0.086–0.117
Baseline Intervention	0.119	0.108–0.130
3-month Control	0.107	0.089–0.124
3-month Intervention	0.120	0.106–0.134
6-month Control	0.109	0.082–0.136
6-month Intervention	0.120	0.105–0.135
9-month Control	0.100	0.084–0.115
9-month Intervention	0.106	0.095–0.117
12-month Control	0.099	0.085–0.113
12-month Intervention	0.112	0.097–0.127
**Campus domain**		
Baseline Control	0.157	0.133–0.182
Baseline Intervention	0.151	0.134–0.167
3-month Control	0.159	0.129–0.190
3-month Intervention	0.193	0.172–0.215
6-month Control	0.154	0.135–0.173
6-month Intervention	0.204	0.177–0.232
9-month Control	0.141	0.122–0.161
9-month Intervention	0.180	0.156–0.203
12-month Control	0.152	0.128–0.176
12-month Intervention	0.205	0.178–0.232
**Neighborhood domain**		
Baseline Control	0.108	0.088–0.129
Baseline Intervention	0.123	0.103–0.143
3-month Control	0.106	0.080–0.131
3-month Intervention	0.184	0.158–0.211
6-month Control	0.092	0.057–0.126
6-month Intervention	0.148	0.126–0.170
9-month Control	0.113	0.081–0.144
9-month Intervention	0.133	0.110–0.156
12-month Control	0.124	0.079–0.169
12-month Intervention	0.149	0.123–0.175
**Beyond neighborhood domain**		
Baseline Control	0.115	0.094–0.136
Baseline Intervention	0.160	0.136–0.184
3-month Control	0.115	0.093–0.136
3-month Intervention	0.242	0.208–0.276
6-month Control	0.129	0.108–0.151
6-month Intervention	0.175	0.144–0.206
9-month Control	0.133	0.101–0.165
9-month Intervention	0.167	0.143–0.192
12-month Control	0.114	0.093–0.135
12-month Intervention	0.175	0.152–0.199

**Appendix 6.** Regression estimates for time spent walking in each domain (min/day)

	Total walk minutes at home	Total walk minutes on campus	Total walk minutes in neighborhood	Total walk minutes beyond neighborhood				
Intervention condition	−1.40	[−25.3,22.6]	−12.93*	[−22.4,−3.5]	−1.86	[−6.6,2.9]	5.88	[−2.9,14.6]
Time point (Ref)								
3 mos	−8.16*	[−14.6,−1.7]	1.49	[−4.9,7.9]	1.57	[−2.5,5.7]	3.60	[−4.8,12.0]
6 mos	−6.66*	[−10.8,−2.5]	4.12	[−1.4,9.6]	−0.20	[−2.4,2.01	0.16	[−3.0,3.3]
9 mos	−7.08*	[−11.1,−3.0]	0.81	[−3.2,4.8]	1.14	[−2.7,5.0]	4.42	[−1.5,10.3]
12 mos	−7.31*	[−11.3,−3.4]	−0.21	[−3.9,3.5]	0.55	[−3.3,4.4]	2.78	[−1.6,7.2]
Condition x time								
Intervention x 3	10.79*	[3.0,18.6]	10.18*	[2.3,18.1]	3.65	[−1.3,8.6]	−2.28	[−11.3,6.7]
Intervention x 6	2.71	[−3.6,9.0]	4.83	[−2.2,11.9]	3.19	[−0.2,6.5]	−0.64	[−4.9,3.7]
Intervention x 9	0.99	[−4.8,6.8]	5.10	[−0.7,10.9]	2.57	[−2.7,7.8]	−6.73*	[−13.2,−0.2]
Intervention x 12	3.04	[−3.5,9.6]	5.49	[−0.1,11.1]	−0.42	[−4.7,3.9]	1.13	[−4.6,6.9]

95% confidence intervals in brackets.

* *p* < 0.05.

**Appendix 7.** Marginal estimates and 95% confidence intervals for time spent walking (min/day) in life-space domains

Time Point x Condition	Marginal estimate (min/day)	95% CI
**Home domain**		
Baseline Control	42.112	37.115–47.108
Baseline Intervention	42.779	37.350–48.208
3-month Control	34.052	27.550–40.554
3-month Intervention	44.701	38.320–51.082
6-month Control	35.225	30.549–39.901
6-month Intervention	37.927	31.556–44.298
9-month Control	34.801	29.945–39.657
9-month Intervention	35.569	30.346–40.793
12-month Control	34.450	29.495–39.405
12-month Intervention	37.754	31.707–43.800
**Campus domain**		
Baseline Control	22.944	19.294–26.594
Baseline Intervention	19.234	16.155–22.312
3-month Control	25.196	18.139–32.252
3-month Intervention	29.967	25.551–34.384
6-month Control	27.375	21.761–32.988
6-month Intervention	26.869	22.694–31.044
9-month Control	23.773	19.205–28.340
9-month Intervention	23.861	20.130–27.593
12-month Control	23.697	19.200–28.194
12-month Intervention	23.732	19.854–27.610
**Neighborhood domain**		
Baseline Control	6.996	4.516–9.476
Baseline Intervention	9.515	7.412–11.618
3-month Control	8.127	4.721–11.533
3-month Intervention	15.893	13.007–18.779
6-month Control	6.074	2.598–9.551
6-month Intervention	14.444	11.456–17.432
9-month Control	6.796	3.948–9.645
9-month Intervention	15.176	11.731–18.621
12-month Control	6.424	3.851–8.997
12-month Intervention	10.842	8.164–13.520
**Beyond neighborhood domain**		
Baseline Control	11.055	8.470–13.640
Baseline Intervention	14.727	12.063–17.391
3-month Control	14.980	6.528–23.433
3-month Intervention	16.893	13.911–19.876
6-month Control	10.962	7.486–14.438
6-month Intervention	15.193	11.926–18.460
9-month Control	14.534	10.163–18.904
9-month Intervention	13.523	10.603–16.444
12-month Control	13.562	10.152–16.971
12-month Intervention	19.190	14.969–23.412

## References

[R1] Abraham A, Sommerhalder K, Abel T, 2010. Landscape and well-being: a scoping study on the health-promoting impact of outdoor environments. Int. J. Publ. Health 10.1007/s00038-009-0069-z. 19768384

[R2] Amaducci L, Maggi S, Langlois J, Minicuci N, Baldereschi M, Di Carlo A, Grigoletto F, Italian Longitudinal Study on Aging Group, 1998. Education and the risk of physical disability and mortality among men and women aged 65 to 84: the Italian longitudinal study on aging ∣ the journals of gerontology: series A ∣ oxford academic. J. Gerontol. Ser. A 53A. M484–M490. 10.1093/gerona/53a.6.m484 9823754

[R3] Amtmann D, Cook KF, Jensen MP, Chen WH, Choi S, Revicki D, Cella D, Rothrock N, Keefe F, Callahan L, Lai JS, 2010. Development of a PROMIS item bank to measure pain interference. Pain 150, 173–182. 10.1016/j.pain.2010.04.025. 20554116 PMC2916053

[R4] Anton SD, Manini TM, Milsom VA, Dubyak P, Cesari M, Cheng J, Daniels MJ, Marsiske M, Pahor M, Leeuwenburgh C, Perri MG, 2011. Effects of a weight loss plus exercise program on physical function in overweight, older women: a randomized controlled trial. Clin. Interv. Aging 6, 141–149. 10.2147/cia.s17001. 21753869 PMC3131984

[R5] Austin PC, Stuart EA, 2015. Moving towards best practice when using inverse probability of treatment weighting (IPTW) using the propensity score to estimate causal treatment effects in observational studies. Stat. Med 34, 3661–3679. 10.1002/sim.6607. 26238958 PMC4626409

[R6] Baker PS, Bodner EV, Allman RM, 2003. Measuring life-space mobility in community-dwelling older adults. J. Am. Geriatr. Soc 51, 1610–1614. 10.1046/j.1532-5415.2003.51512.x. 14687391

[R7] Bandinelli S, Lauretani F, Boscherini V, Gandi F, Pozzi M, Corsi AM, Bartali B, Lova RM, Guralnik JM, Ferrucci L, 2006. A randomized, controlled trial of disability prevention in frail older patients screened in primary care: the FRASI study. Design and baseline evaluation. Aging Clin. Exp. Res 18, 359–366. 10.1007/BF03324831. 17167299 PMC2659809

[R8] Bandura A, 1986. Social Foundations of Thought and Action: A Social Cognitive Theory. Prentice Hall, Englewood Cliffs, NJ.

[R9] Barha CK, Davis JC, Falck RS, Nagamatsu LS, Liu-Ambrose T, 2017. Sex differences in exercise efficacy to improve cognition: a systematic review and meta-analysis of randomized controlled trials in older humans. Front. Neuroendocrinol 10.1016/j.yfrne.2017.04.002. 28442274

[R10] Barnett DW, Barnett A, Nathan A, Van Cauwenberg J, Cerin E, 2017. Built environmental correlates of older adults’ total physical activity and walking: a systematic review and meta-analysis. Int. J. Behav. Nutr. Phys. Activ 10.1186/s12966-017-0558-z. PMC5547528 28784183

[R11] Beauchamp MK, Schmidt CT, Pedersen MM, Bean JF, Jette AM, 2014. Psychometric properties of the late-life function and disability instrument: a systematic review. BMC Geriatr. 10.1186/1471-2318-14-12. PMC3909447 24476510

[R12] Béland F, Julien D, Bier N, Desrosiers J, Kergoat M-J, Demers L, 2018. Association between cognitive function and life-space mobility in older adults: results from the FRéLE longitudinal study. BMC Geriatr. 18 10.1186/s12877-018-0908-y. PMC6154880 30249199

[R13] Boruff BJ, Nathan A, Nijënstein S, 2012. Using GPS technology to (re)-examine operational definitions of “neighbourhood” in place-based health research. Int. J. Health Geogr 11, 1–14. 10.1186/1476-072X-11-22. 22738807 PMC3490929

[R14] Brawley L, Rjeski J, King A, 2003. Promoting physical activity for older adults The challenges for changing behavior. Am. J. Prev. Med 25, 172–183. 10.1016/S0749-3797(03)00182-X. 14552942

[R15] Brookhart MA, Schneeweiss S, Sturmer T, 2006. Variable selection for propensity score models. Am. J. Epidemiol 163, 1149–1156. 16624967 10.1093/aje/kwj149 PMC1513192

[R16] Carlson JA, Jankowska MM, Meseck K, Godbole S, Natarajan L, Raab F, Demchak B, Patrick K, Kerr J, 2015. Validity of PALMS GPS scoring of active and passive travel compared with SenseCam. Med. Sci. Sports Exerc 47, 662–667. 10.1249/MSS.0000000000000446. 25010407 PMC4289119

[R17] Chen F-T, Etnier J, Wu C-H, Cho Y-M, Hung T-M, Chang Y-K, 2018. Dose-Response relationship between exercise duration and executive function in older adults. J. Clin. Med 7, 279. 10.3390/jcm7090279. PMC6162829 30217031

[R18] Choi L, Liu Z, Matthews CE, Buchowski MS, 2011. Validation of accelerometer wear and nonwear time classification algorithm. Med. Sci. Sports Exerc 43, 357–364. 10.1249/MSS.0b013e3181ed61a3. 20581716 PMC3184184

[R19] Consoli A, Nettel-Aguirre A, Spence JC, McHugh TL, Mummery K, McCormack GR, 2020. Associations between objectively-measured and self-reported neighbourhood walkability on adherence and steps during an internet-delivered pedometer intervention. PloS One 15, e0242999. 10.1371/journal.pone.0242999. 33270692 PMC7714347

[R20] De Silva NA, Gregory MA, Venkateshan SS, Verschoor CP, Kuspinar A, 2019. Examining the association between life-space mobility and cognitive function in older adults: a systematic review. J. Aging Res 10.1155/2019/3923574. PMC6589294 31275650

[R21] Dyck D. Van, Cerin E, Conway TL, De Bourdeaudhuij I, Owen N, Kerr J, Cardon G, Frank LD, Saelens BE, Sallis JF, 2012. Perceived neighborhood environmental attributes associated with adults’ transport-related walking and cycling: findings from the USA, Australia and Belgium. Int. J. Behav. Nutr. Phys. Activ 9 10.1186/1479-5868-9-70. PMC3489620 22691723

[R22] Fillekes MP, Röcke C, Katana M, Weibel R, 2019. Self-reported versus GPS-derived indicators of daily mobility in a sample of healthy older adults. Soc. Sci. Med 220, 193–202. 10.1016/j.socscimed.2018.11.010. 30453111

[R23] Gallagher NA, Clarke PJ, Gretebeck KA, 2014. Gender differences in neighborhood walking in older adults. J. Aging Health 26, 1280–1300. 10.1177/0898264314532686. 25502242

[R24] Guralnik JM, Ferrucci L, Pieper CF, Leveille SG, Markides KS, Ostir GV, Studenski S, Berkman LF, Wallace RB, 2000. Lower extremity function and subsequent disability: consistency across studies, predictive models, and value of gait speed alone compared with the short physical performance battery. J. Gerontol. - Ser. A Biol. Sci. Med. Sci 55 10.1093/gerona/55.4.M221. PMC12149745 10811152

[R25] Guralnik JM, Simonsick EM, Ferrucci L, Glynn RJ, Berkman LF, Blazer DG, Scherr PA, Wallace RB, 1994. A short physical performance battery assessing lower extremity function: association with self-reported disability and prediction of mortality and nursing home admission. J. Gerontol 49 10.1093/geronj/49.2.M85. 8126356

[R26] Hirsch JA, Winters M, Ashe MC, Clarke P, McKay H, 2016. Destinations that older adults experience within their GPS activity spaces relation to objectively measured physical activity. Environ. Behav 48, 55–77. 10.1177/0013916515607312. 26783370 PMC4714356

[R27] Hirsch JA, Winters M, Clarke P, McKay H, 2014. Generating GPS activity spaces that shed light upon the mobility habits of older adults: a descriptive analysis. Int. J. Health Geogr 13 10.1186/1476-072X-13-51. PMC4326206 25495710

[R28] James BDB, Boyle P. a, Buchman AAS, Barnes LL, Bennett D. a, 2011. Life space and risk of Alzheimer disease, mild cognitive impairment, and cognitive decline in old age. Am. J 19, 961–969. 10.1097/JGP.0b013e318211c219.Life. PMC3123692 21430509

[R29] Jansen CP, Diegelmann M, Schilling OK, Werner C, Schnabel EL, Wahl HW, Hauer K, 2018. Pushing the boundaries: a physical activity intervention extends sensor-assessed life-space in nursing home residents. Gerontol. 58, 979–988. 10.1093/geront/gnx136. 28958082

[R30] Johnson J, Rodriguez MA, Snih S. Al, 2020. Life-space mobility in the elderly: current perspectives. Clin. Interv. Aging 15, 1665–1674. 10.2147/CIA.S196944. 32982200 PMC7501960

[R31] Kempen GIJM, Yardley L, Van Haastregt JCM, Zijlstra GAR, Beyer N, Hauer K, Todd C, 2008. The Short FES-I: a shortened version of the falls efficacy scale-international to assess fear of falling. Age Ageing 37, 45–50. 10.1093/ageing/afm157. 18032400

[R32] Kerr J, Carlson J.a., Sallis JF, Rosenberg D, Leak CR, Saelens BE, Chapman JE, Frank LD, Cain KL, Conway TL, King AC, 2011. Assessing health-related resources in senior living residences. J. Aging Stud 25, 206–214. 10.1016/j.jaging.2011.03.004. 27168700 PMC4860260

[R33] Kerr J, Marshall S, Godbole S, Neukam S, Crist K, Wasilenko K, Golshan S, Buchner D, 2012a. The relationship between outdoor activity and health in older adults using GPS. Int. J. Environ. Res. Publ. Health 9, 4615–4625. 10.3390/ijerph9124615. PMC3546779 23330225

[R34] Kerr J, Norman GJ, Adams M.a., Ryan S, Frank L, Sallis JF, Calfas KJ, Patrick K, 2010. Do neighborhood environments moderate the effect of physical activity lifestyle interventions in adults? Health Place 16, 903–908. 10.1016/j.healthplace.2010.05.002. 20510642 PMC2918657

[R35] Kerr J, Rosenberg D, Millstein RA, Bolling K, Crist K, Takemoto M, Godbole S, Moran K, Natarajan L, Castro-Sweet C, Buchner D, 2018. Cluster randomized controlled trial of a multilevel physical activity intervention for older adults. Int. J. Behav. Nutr. Phys. Activ 15 10.1186/s12966-018-0658-4. PMC5879834 29609594

[R36] Kerr J, Rosenberg D, Nathan A, Millstein R. a, Carlson JA, Crist K, Wasilenko K, Bolling K, Castro CM, Buchner DM, Marshall S, 2012b. Applying the ecological model of behavior change to a physical activity trial in retirement communities: description of the study protocol. Contemp. Clin. Trials 33, 1180–1188. 10.1016/j.cct.2012.08.005.Applying. 22921641 PMC3468706

[R37] Kerr J, Sallis JF, Saelens BE, Cain KL, Conway TL, Frank LD, King AC, 2012c. Outdoor physical activity and self rated health in older adults living in two regions of the U.S. Int. J. Behav. Nutr. Phys. Activ 9, 89. 10.1186/1479-5868-9-89. PMC3464785 22846594

[R38] King AC, Sallis JF, Frank LD, Saelens BE, Cain K, Conway TL, Chapman JE, Ahn DK, Kerr J, 2011. Aging in neighborhoods differing in walkability and income: associations with physical activity and obesity in older adults. Soc. Sci. Med 73, 1525–1533. 10.1016/J.SOCSCIMED.2011.08.032. 21975025 PMC3637547

[R39] Kuspinar A, Verschoor CP, Beauchamp MK, Dushoff J, Ma J, Amster E, Bassim C, Dal Bello-Haas V, Gregory MA, Harris JE, Letts L, Neil-Sztramko SE, Richardson J, Valaitis R, Vrkljan B, 2020. Modifiable factors related to life-space mobility in community-dwelling older adults: results from the Canadian Longitudinal Study on Aging. BMC Geriatr. 20, 35. 10.1186/s12877-020-1431-5. 32005107 PMC6995110

[R40] Lam MS, Godbole S, Chen J, Oliver M, Badland H, Marshall S, Kelly P, Foster C, Doherty A, Kerr J, 2013. Measuring time spent outdoors using a wearable camera and GPS. In: Proceedings of the 4th International SenseCam & Pervasive Imaging Conference, pp. 1–7.

[R41] Leveille SG, Penninx BWJH, Melzer D, Izmirlian G, Guralnik’ JM, 2000. Sex differences in the prevalence of mobility disability in old age: the dynamics of incidence, recovery, and mortality. J. Gerontol.: Soc. Sci 55B (1), S41–S50. 10.1093/geronb/55.1.s41 10728129

[R42] Leyrat C, Caille A, Donner A, Giraudeau B, 2013. Propensity scores used for analysis of cluster randomized trials with selection bias: a simulation study. Stat. Med 32, 3357–3372. 10.1002/sim.5795. 23553813

[R43] Liddle J, Ireland D, McBride SJ, Brauer SG, Hall LM, Ding H, Karunanithi M, Hodges PW, Theodoros D, Silburn PA, Chenery HJ, 2014. Measuring the lifespace of people with Parkinson’s disease using smartphones: proof of principle. J. Med. Internet Res 16 10.2196/mhealth.2799. PMC4114414 25100206

[R44] Lo BK, Graham ML, Folta SC, Paul LC, Strogatz D, Nelson ME, Parry SA, Carfagno ME, Wing D, Higgins M, Seguin RA, 2019. Examining the associations between walk score, perceived built environment, and physical activity behaviors among women participating in a community-randomized lifestyle change intervention trial: strong hearts, healthy communities. Int. J. Environ. Res. Publ. Health 16. 10.3390/ijerph16050849. PMC6427661 30857189

[R45] Mackey DC, Cauley JA, Barrett-Connor E, Schousboe JT, Cawthon PM, Cummings SR, 2014. Life-space mobility and mortality in older men: a prospective cohort study. J. Am. Geriatr. Soc 62, 1288–1296. 10.1111/jgs.12892. 24934163 PMC4251711

[R46] Mackey DC, Lui LY, Cawthon PM, Ensrud K, Yaffe K, Cummings SR, 2016. Life-space mobility and mortality in older women: prospective results from the study of osteoporotic fractures. J. Am. Geriatr. Soc 64, 2226–2234. 10.1111/jgs.14474. 27696354 PMC5118116

[R47] Matthews C, 2005. Calibration of accelerometer output for adults. Med. Sci. Sports Exerc 37, S512–S522. 10.1249/01.mss.0000185659.11982.3d. 16294114

[R48] Matthews CE, Keadle SK, Sampson J, Lyden K, Bowles HR, Moore SC, Libertine A, Freedson PS, Fowke JH, 2013. Validation of a previous-day recall measure of active and sedentary behaviors. Med. Sci. Sports Exerc 45, 1629–1638. 10.1249/MSS.0b013e3182897690. 23863547 PMC3717193

[R49] Matthews RJ, Smith LK, Hancock RM, Jagger C, Spiers NA, 2005. Socioeconomic factors associated with the onset of disability in older age: a longitudinal study of people aged 75 years and over. In: Social Science and Medicine. Soc Sci Med, pp. 1567–1575. 10.1016/j.socscimed.2005.02.007. 16005788

[R50] Matthews SA, Yang TC, 2013. Spatial polygamy and contextual exposures (SPACEs): promoting activity space approaches in research on place and health. Am. Behav. Sci 57, 1057–1081. 10.1177/0002764213487345. 24707055 PMC3975622

[R51] Mavoa S, Bagheri N, Koohsari MJ, Kaczynski AT, Lamb KE, Oka K, O’Sullivan D, Witten K, 2019. How do neighbourhood definitions influence the associations between built environment and physical activity? Int. J. Environ. Res. Publ. Health 16, 1501. 10.3390/ijerph16091501. PMC6540146 31035336

[R52] May D, Nayak USL, Isaacs B, 1985. The life-space diary: a measure of mobility in old people at home. Disabil. Rehabil 7, 182–186. 10.3109/03790798509165993. 4093250

[R53] Merom D, Gebel K, Fahey P, Astell-Burt T, Voukelatos A, Rissel C, Sherrington C, 2015. Neighborhood walkability, fear and risk of falling and response to walking promotion: the Easy Steps to Health 12-month randomized controlled trial. Prev. Med. Reports 2, 704–710. 10.1016/j.pmedr.2015.08.011. PMC4721485 26844140

[R54] Meseck K, Jankowska MM, Schipperijn J, Natarajan L, Godbole S, Carlson JA, Takemoto M, Crist K, Kerr J, 2016. Is missing geographic position system (GPS) data in accelerometry studies a problem, and is imputation the solution? Geospat. Heal 11, 157–163. 10.4081/gh.2016.403. PMC4964846 27245796

[R55] Moerbeek M, 2005. Randomization of clusters versus randomization of persons within clusters: which is preferable? Am. Statistician 59, 72–78. 10.1198/000313005X20727.

[R56] Moerbeek M, Van Schie S, 2016. How large are the consequences of covariate imbalance in cluster randomized trials: a simulation study with a continuous outcome and a binary covariate at the cluster level. BMC Med. Res. Methodol 16 10.1186/s12874-016-0182-7. PMC4939594 27401771

[R57] Peel C, Baker PS, Roth DL, Brown CJ, Bodner EV, Allman RM, 2005. Assessing mobility in older adults: the UAB study of aging life-space assessment. Phys. Ther 85, 1008–1019. 10.1093/ptj/85.10.1008. 16180950

[R58] Perez LG, Kerr J, Sallis JF, Slymen D, McKenzie TL, Elder JP, Arredondo EM, 2018. Perceived neighborhood environmental factors that maximize the effectiveness of a multilevel intervention promoting physical activity among latinas. Am. J. Health Promot 32, 334–343. 10.1177/0890117117742999. 29166779 PMC6645781

[R59] Portegijs E, Rantakokko M, Mikkola TM, Viljanen A, Rantanen T, 2014. Association between physical performance and sense of autonomy in outdoor activities and life-space mobility in community-dwelling older people. J. Am. Geriatr. Soc 62, 615–621. 10.1111/jgs.12763. 24655124

[R60] Portegijs E, Tsai L-T, Rantanen T, Rantakokko M, 2015. Moving through Life-Space Areas and Objectively Measured Physical Activity of Older People. 10.1371/journal.pone.0135308. PMC4529301 26252537

[R61] Rantakokko M, Iwarsson S, Vahaluoto S, Portegijs E, Viljanen A, Rantanen T, 2014. Perceived environmental barriers to outdoor mobility and feelings of loneliness among community-dwelling older people. J. Gerontol. - Ser. A Biol. Sci. Med. Sci 69, 1562–1568. 10.1093/gerona/glu069. 24864307

[R62] Rantakokko M, Mänty M, Iwarsson S, Törmäkangas T, Leinonen R, Heikkinen E, Rantanen T, 2009. Fear of moving outdoors and development of outdoor walking difficulty in older people: clinical Investigations. J. Am. Geriatr. Soc 57, 634–640. 10.1111/j.1532-5415.2009.02180.x. 19392955

[R63] Rejeski WJ, Marsh AP, Brubaker PH, Buman M, Fielding RA, Hire D, Manini T, Rego A, Miller ME, 2016. Analysis and interpretation of accelerometry data in older adults: the LIFE study. J. Gerontol. - Ser. A Biol. Sci. Med. Sci 71, 521–528. 10.1093/gerona/glv204. 26515258 PMC5175451

[R64] Rosenbaum PR, Rubin DB, 1983. The central role of the propensity score in observational studies for causal effects. Biometrika 70, 41–55.

[R65] Sallis JF, Cervero RB, Ascher W, Henderson KA, Kraft MK, Kerr J, 2006. An ecological approach to creating active living communities. Annu. Rev. Publ. Health 27, 297–322. 10.1146/annurev.publhealth.27.021405.102100. 16533119

[R66] Sanders LMJ, Hortobágyi T, la Bastide-van Gemert S, Van Der Zee EA, Van Heuvelen MJG, 2019. Dose-response relationship between exercise and cognitive function in older adults with and without cognitive impairment: a systematic review and meta-analysis. PloS One. 10.1371/journal.pone.0210036. PMC6328108 30629631

[R67] Sayers SP, Jette AM, Haley SM, Heeren TC, Guralnik JM, Fielding RA, 2004. Validation of the late-life function and disability instrument. J. Am. Geriatr. Soc 52, 1554–1559. 10.1111/j.1532-5415.2004.52422.x. 15341561

[R68] Schenk AK, Witbrodt BC, Hoarty CA, Carlson RH, Goulding EH, Potter JF, Bonasera SJ, 2011. Cellular telephones measure activity and lifespace in community-dwelling adults: proof of principle. J. Am. Geriatr. Soc 59, 345–352. 10.1111/j.1532-5415.2010.03267.x. 21288235 PMC3056384

[R69] Shah R, Maitra K, Barnes L, James B, Leurgans S, Bennett D, 2012. Relation of driving status to incident life space constriction in community-dwelling older persons: a prospective cohort study. J. Gerentol. A Biol. Sci. Med. Sci 67, 984–989. 10.1093/gerona/gls133. PMC3436089 22546958

[R70] Shareck M, Frohlich KL, Kestens Y, 2014. Considering daily mobility for a more comprehensive understanding of contextual effects on social inequalities in health: a conceptual proposal. Health Place 29, 154–160. 10.1016/j.healthplace.2014.07.007. 25103785

[R71] Shoval N, Wahl H-W, Auslander G, Isaacson M, Oswald F, Edry T, Landau R, Heinik J, 2011. Use of the global positioning system to measure the out-of-home mobility of older adults with differing cognitive functioning. Ageing Soc. 31, 849–869. 10.1017/S0144686X10001455.

[R72] Stalvey BT, Owsley C, Sloane ME, Ball K, 1999a. The life space questionnaire: a measure of the extent of mobility of older adults. J. Appl. Gerontol 18, 460–478. 10.1177/073346489901800404.

[R73] Stalvey BT, Owsley C, Sloane ME, Ball K, 1999b. The life space questionnaire: a measure of the extent of mobility of older adults. J. Appl. Gerontol 18, 460–478. 10.1177/073346489901800404.

[R74] Takemoto M, Carlson JA, Moran K, Godbole S, Crist K, Kerr J, 2015. Relationship between objectively measured transportation behaviors and health characteristics in older adults. Int. J. Environ. Res. Publ. Health 12. 10.3390/ijerph121113923. PMC4661624 26528999

[R75] Taylor JK, Buchan IE, van der Veer SN, 2019. Assessing life-space mobility for a more holistic view on wellbeing in geriatric research and clinical practice. Aging Clin. Exp. Res 10.1007/s40520-018-0999-5. PMC6439151 30078096

[R76] Tsai LT, Portegijs E, Rantakokko M, Viljanen A, Saajanaho M, Eronen J, Rantanen T, 2015. The association between objectively measured physical activity and life-space mobility among older people. Scand. J. Med. Sci. Sports 25, e368–e373. 10.1111/sms.12337. 26152855

[R77] Van Dyck D, Cerin E, Conway TL, De Bourdeaudhuij I, Owen N, Kerr J, Cardon G, Frank LD, Saelens BE, Sallis JF, 2012. Perceived neighborhood environmental attributes associated with adults’ transport-related walking and cycling: findings from the USA, Australia and Belgium. Int. J. Behav. Nutr. Phys. Activ 9, 1–14. 10.1186/1479-5868-9-70. PMC3489620 22691723

[R78] Vestergaard S, Patel KV, Bandinelli S, Ferrucci L, Guralnik JM, 2009. Characteristics of 400-meter walk test performance and subsequent mortality in older adults. Rejuvenation Res. 12, 177–184. 10.1089/rej.2009.0853. 19594326 PMC2939839

[R79] Vidoni ED, Johnson DK, Morris JK, Van Sciver A, Greer CS, Billinger SA, Donnelly JE, Burns JM, 2015. Dose-response of aerobic exercise on cognition: a community-based, pilot randomized controlled trial. PloS One 10. 10.1371/journal.pone.0131647. PMC4497726 26158265

[R80] Wahl H-W, Wettstein M, Shoval N, Oswald F, Kaspar R, Issacson M, Voss E, Auslander G, Heinik J, 2013. Interplay of cognitive and motivational resources for out-of-home behavior in a sample of cognitively heterogeneous older adults: findings of the sentra project interplay of cognitive and motivational resources for out-of-home behavior in a sample of cognitively heterogeneous older adults: findings of the SenTra project. J. Gerontol. B Psychol. Sci. Soc. Sci 68, 691–702. 10.1093/geronb/gbs106. 23197344

[R81] Wahl H-W, Wettstein M, Shoval N, Oswald F, Kaspar R, Issacson M, Voss E, Auslander G, Heinik J, 2013. Interplay of cognitive and motivational resources for out-of-home behavior in a sample of cognitively heterogeneous older adults: findings of the SenTra project. J. Gerontol. B Psychol. Sci. Soc. Sci 68, 691–702. 10.1093/geronb/gbs106. 23197344

[R82] Wan N, Qu W, Whittington J, Witbrodt BC, Henderson MP, Goulding EH, Schenk AK, Bonasera SJ, Lin G, 2013. Assessing smart phones for generating life-space indicators. Environ. Plann. Plann. Des 40, 350–361. 10.1068/b38200. PMC5556372 28819332

[R83] Webber SC, Porter MM, Menec VH, 2010. Mobility in older adults: a comprehensive framework. Gerontol. 50, 443–450. 10.1093/geront/gnq013. 20145017

[R84] Williams R, 2012. Using the margins command to estimate and interpret adjusted predictions and marginal effects. STATA J. 12, 308–331. 10.1177/1536867x1201200209.

[R85] Winters M, Voss C, Ashe MC, Gutteridge K, McKay H, Sims-Gould J, 2014. Where do they go and how do they get there? Older adults’ travel behaviour in a highly walkable environment. Soc. Sci. Med 133, 304–312. 10.1016/j.socscimed.2014.07.006. 25017579

[R86] World Population Ageing 2019 Highlights, 2019.

[R87] Yen IH, Shim JK, Martínez AD, 2012. Application of two schools of social theory to neighbourhood, place and health research. In: O’Campo P DJ (Ed.), Rethinking Social Epidemiology: towards a Science of Change, pp. 157–174.

[R88] Zlatar ZZ, Godbole S, Takemoto M, Crist K, Sweet CMC, Kerr J, Rosenberg DE, 2019. Changes in moderate intensity physical activity are associated with better cognition in the multilevel intervention for physical activity in retirement communities (MIPARC) study. Am. J. Geriatr. Psychiatr 27, 1110–1121. 10.1016/j.jagp.2019.04.011. PMC6739142 31138456

